# Supplementary material for: Species Diversity and Ecological Habitat of Absidia (Cunninghamellaceae, Mucorales) with Emphasis on Five New Species from Forest and Grassland Soil in China
Source: J Fungi (Basel). 2022 Apr 30;8(5):471. doi: 10.3390/jof8050471 (PMC9146633; doi:10.3390/jof8050471)
Supplement: Supplementary file 1 [file jof-08-00471-s001.zip › jof-1696872-supplementary/Supplementary Table S1.pdf]

Supplementary Table S1. Top hits for the new species based on BLAST search for ITS sequences from type materials.

Query	Sbjct	Query cover (%)	Percent identity (%)
Absidia abundans	*A. panacisoli* CBS 140959	96	93.90
	A. panacisoli SYPF 7183	96	93.90
	A. anomala CBS 125.68	47	91.63
	A. caatinguensis URM 7156	38	95.69
	A. healeyae UoMAU1	39	95.28
A. lobata	*A. glauca* CBS 101.08	40	92.00
	A. californica	32	94.89
	A. fusca CBS 102.35	36	91.92
	A. cornuta URM 6100	36	90.59
	A. pseudocylindrospora CBS 100.62	36	90.59
A. radiata	*A. montepascoalis* URM 8218	76	85.14
	A. anomala CBS 125.68	61	89.15
	A. caatinguensis URM 7156	72	83.80
	A. edaphica MFLUCC 20-0088	73	83.76
	A. soli MFLUCC 20-0089	50	91.18
A. yunnanensis	*A. montepascoalis* URM 8218	90	81.92
	A. anomala CBS 125.68	64	85.52
	A. caatinguensis URM 7156	62	85.63
	A. edaphica MFLUCC 20-0088	63	84.83
	A. soli MFLUCC 20-0089	61	84.94
A. sichuanensis	*A. psychrophilia* CBS 128.68	94	92.18
	A. healeyae UoMAU1	63	86.10
	A. cylindrospora var. *nigra* CBS 127.68	45	92.31
	A. edaphica MFLUCC 20-0088	45	91.44
	A. pernambucoensis URM:7219	44	91.34
